# The pathophysiological role of bacterial biofilms in chronic sinusitis

**DOI:** 10.1007/s00405-015-3650-5

**Published:** 2015-05-30

**Authors:** Jolanta Dlugaszewska, Malgorzata Leszczynska, Marcin Lenkowski, Agnieszka Tatarska, Tomasz Pastusiak, Witold Szyfter

**Affiliations:** Department of Genetics and Pharmaceutical Microbiology, Karol Marcinkowski University of Medical Sciences, Ul. Przybyszewskiego 49, Poznan, Poland; Department of Otolaryngology, Head and Neck Surgery, Poznan University of Medical Sciences, Poznan, Poland

**Keywords:** Bacterial biofilm, Sinusitis, Microflora

## Abstract

Chronic rhinosinusitis (CRS) is a very common disorder that remains poorly understood from a pathogenic standpoint. Recent research on the pathogenesis of CRS has been focused on the potential role of biofilms in this chronic infection. The aim of this study was to assess the sinuses’ microflora and biofilm formation on the sino-nasal mucosa in patients with CRS. Paranasal sinus mucosa specimens were harvested at the time of functional endoscopic sinus surgery (FESS). Classical microbiology techniques for the isolation and identification of sinus mucosa microbial flora were used. Scanning electron microscopy (SEM) was used to detect biofilm on the surface of mucosa. A microtiter plate assay for in vitro biofilm formation was employed, divided into three aliquots. One part was assessed for bacterial presence, utilizing an API manual system and the Vitek^®^ 2 Compact system. The two remaining aliquots were tested by in vitro conventional microbiological assay with the use of the Infinite M200 (Tecan) microtiter plate reader, and also by scanning electron microscopy (SEM). A microbiological examination of mucosal specimens had taken during FESS operation revealed the presence of various types of bacteria in 29 out of 30 tested samples. Out of 62 different strains isolated from patients with CRS, 23 strains of coagulase-negative *Staphylococcus epidermidis* and 6 strains of *Escherichia coli* were the most frequently isolated microorganisms, accounting for 37.1 and 9.7 %, respectively. Among the 62 isolated strains, 58 were used to assess biofilm formation. From the total of 58 isolates, 8.6 % were strong biofilm producers, 20.7 % were moderate, and 70.7 % of isolates were considered to be non- or weak biofilm producers. SEM of the 30 nasal concha mucosal samples taken from patients with CRS revealed biofilm in 23 specimens. A marked destruction of the epithelium was observed, with variation in degrees of severity, from disarrayed cilia to complete absence of cilia. The vast majority of nasal concha mucosal samples of patients affected by chronic sinusitis presented with biofilm formation. Our study showed that 76.7 % of patients having FESS for CRS had evidence of biofilms on SEM micrographs. Although certain detection methods could lead to various discrepancies in the amount of biofilm produced, the consistent demonstration of biofilms in patients with CRS suggests that this convoluted three-dimensional structures might play a significant role in either the pathogenesis or persistence of chronic rhinosinusitis.

## Introduction

Chronic rhinosinusitis (CRS) is one of the most common chronic disorders, affecting 4–28 % of the European and US populations. This disease significantly reduces the quality-of-life of its sufferers and is a socioeconomic burden on the community. Patients with recurrent or chronic rhinosinusitis report a deteriorating sense of general health and vitality when compared to the general population. CRS represents a spectrum of inflammatory and infectious processes concurrently affecting the nose and paranasal sinuses and is characterized by a minimum of two symptoms. These include nasal congestion or nasal discharge (anterior/posterior nasal drip), facial pain, and a reduction in the sense of smell. In addition, the presence of polyps and mucosal edema is one of the main presentations at examination. The duration of the disease tends to exceed 12 weeks [1].

Chronic sinusitis may or may not involve polyps. Maxillary sinus ostia, anterior ethmoidal cells and their ostia, ethmoid infundibulum, hiatus semilunaris and middle meatus form the osteo-meatal complex which plays a monumental role in the pathogenesis of chronic sinusitis. In an individual with a healthy respiratory tract, the mucocillary clearance and patent ostia allow for the effortless removal of mucosal secretions. Impairment of the osteo-meatal complex or excessive mucosal production increases the chance of the infectious process [2].

In CRS without the involvement of polyps, the cellular infiltration includes neutrophilic granulocytes. This type of inflammation is observed in patients with poor ventilation and impaired sinus drainage. In the cases of chronic sinusitis with polyps, eosinophilic granulocytes predominate as the main inflammatory cells.

CRS represents a heterogeneous group of diseases resulting from the multifaceted interaction between the host and the environment [3, 4].

Despite the fact that bacteria and fungi have been linked to the development of CRS, the nature of their interaction with the host remains largely unknown. It is unclear whether bacteria cause infection, expose the host to superantigens causing an inflammatory response, or are able to colonize due to the pre-existing pathology of the sinus mucosa [5].

The discovery of bacteria existing in an alternative biofilm form has led many researchers to revisit the pathogenesis of sinus disease [6].

Infection in the form of biofilm may have an important, if not central, role in the maintenance of the recalcitrant inflammation for this increasingly common chronic disease.

Now, CRS is thought to have an underlying biofilm etiology. In contrast to the planktonic infections, biofilms are highly capable of evoking sustained responses from the host’s immune system [7, 8].

Biofilm is a three-dimensionally structured, specialized community of adherent microorganisms surrounded by an extracellular polymeric substance (EPS). Biofilm communities in most environments, including human disease, tend to be polymicrobial. By including multiple bacterial and/or fungal species in a single community, biofilms obtain numerous advantages, such as passive resistance, metabolic cooperation, by-product influence, quorum sensing systems, an enlarged gene pool with more efficient DNA sharing, and many other synergies, which give them a competitive advantage. In general, the greater the diversity, the more robust the biofilm is in terms of its survivability [9]. However, knowledge of the importance of bacteria and microbial biofilm in the etiology of CRS is still incomplete and controversial. The aim of this study was to assess sinus microflora and biofilm formation on the sino-nasal mucosa in patients with CRS.

Furthermore, the assessment of the ability of isolated microorganisms to form biofilm in vitro was tested.

## Materials and methods

### Study population

The study was conducted in the ENT (Ear, Nose and Throat) Department of Poznan University of Medical Sciences. It was approved by the local bioethics commission. The study group, comprising 30 patients with chronic sinusitis with nasal polyps undergoing endoscopic sinus surgery, was compared with 20 control patients undergoing septoplasty surgery without sinusitis and polyps.

The study group included adult patients with CRS diagnosed on the basis of their medical history and physical examination, according to the criteria established by the European Position Paper on Rhinosinusitis and Nasal Polyps (EPOS2012) group. The exclusion criteria were immunodeficiency, ciliary dyskinesia, and acute upper respiratory tract infection. Neither the study group nor the control group received antibiotics or steroids in the 4 weeks before surgery.

### Sample collection

Nasal concha mucosa samples were harvested from patients with CRS at the time of functional endoscopic sinus surgery (FESS) and from control patients, at the time of nasal septoplasty and rhinoplasty. Samples of about 5 × 5 mm were taken in triplicate: two samples for microbiological examination, and one for scanning electron microscopy (SEM). The tissue specimens were processed within 2 h of collection.

### Isolation and identification of microorganisms

Tissue specimens were homogenized in 1 ml of 0.9 % NaCl, and 0.1 ml aliquots of the homogenate were inoculated onto blood agar (bioMérieux, France), chocolate haemophilus agar (bioMérieux, France), mannitol salt agar (bioMérieux, France), MacConcey agar (bioMérieux, France), and Sabouraud agar (bioMérieux, France), as well as in tryptic soy broth (bioMérieux, France). For the isolation of anaerobic bacteria, one sample was inoculated directly into Scheadler broth (bioMérieux, France) immediately after collection. Cultures were incubated at 35 ± 1 °C for 24–72 h on solid agar media, and for 7 days in liquid media. Blood agar and chocolate agar plates were incubated in an increased concentration of CO_2_. Turbid broth media were sub-cultured onto a solid agar medium.

Bacteria were identified using conventional microbiological methods, the API manual system (bioMérieux, France), and the Vitek^®^ 2 Compact system (bioMérieux, France).

### Detection of biofilm on nasal concha mucosa

The detection of biofilm was performed by scanning electron microscopy (SEM). Specimens were rinsed with PBS, dehydrated, and fixed in a series of increasing acetone concentrations from 10 to 100 %. The specimens in 100 % acetone were sent to the Laboratory of Electron and Confocal Microscopy, at the Adam Mickiewicz University, Poznan. Following the preparation process, the specimens were examined for the presence of biofilm structures with a ZEISS EVO 40 scanning electron microscope. The images were compared with the available database of biofilm images.

Two randomly selected individuals participated in a blind study in which specimens were examined for the presence of biofilm formation.

### In vitro biofilm formation

The microtiter plate method was utilized as an indicator of biofilm formation. Isolates grown for 24 h on tryptic soy agar (bioMérieux, France) were suspended in a tryptic soy broth (bioMérieux, France) and adjusted to a turbidity of 0.5 MacFarland. Then, 200 µl aliquots of each isolate were placed into 96-well flat-bottom microtiter plates and incubated at 37 ± 1 °C for 20 h. The culture medium was then discarded from the microtiter plate, and the wells were washed three times with deionized water, stained with 0.1 % crystal violet for 15 min, rinsed with water, and air dried overnight. The crystal violet from stained biofilm was resuspended in 250 µl of 95 % ethanol. The optical density (OD) of stained adherent biofilm was measured using an Infinite M200 (Tecan) plate reader at a wavelength of 590 nm. Wells containing uninoculated TSB media served as a negative control. Tests were repeated three times. The data were averaged and standard deviation was calculated.

The interpretation of biofilm formation was done according to the criteria of Stepanovic et al. [10] (Table 1).

**Table 1:** Classification of biofilm formation

OD values	Biofilm formation
≤ODc	Non
2 × ODc ≤ OD > ODc	Weak
4 × ODc ≤ OD > 2 × ODc	Moderate
>4 × ODc	High

*ODC* mean OD of control probes + 3SD

## Results

A microbiological examination of tissue specimens taken from patients with CRS revealed the presence of various types of bacteria in 29 out of 30 studied samples. Eighty percent of samples had mixed flora. In total, 62 different microorganisms were isolated (Table 2). *Staphylococcus epidermidis*, the most frequently found microorganism, and other coagulase-negative *Staphylococcus* (CNS) were present in 86.6 % of samples, and *S. aureus* was present in 23 % of the samples. Gram-negative microorganisms were identified in one third of samples, and anaerobic bacteria were found in 13 % of samples.

**Table 2:** Microorganisms isolated from CRS patients and the control group

Microorganism	*n* (%) study group	*n* (%) control group
Aerobic bacteria		
*Staphylococcus aureus*	7 (11.3)	3 (8.8)
*Staphylococcus epidermidis*	23 (37.1)	14 (41.2)
*Staphylococcus lugdunensis*	1 (1.6)	–
*Staphylococcus capitis*	1 (1.6)	–
*Staphylococcus saprophyticus*	1 (1.6)	6 (17.7)
*Staphylococcus haemolyticus*	5 (8.1)	7 (20.6)
Other CNS	2 (3.2)	1 (2.9)
*Streptococcus mitis*	1 (1.6)	3 (8.8)
*Kocuria rosea*	1 (1.6)	–
*Kocuria kristinae*	1 (1.6)	–
*Enterococcus faecalis*	2 (3.2)	–
*Escherichia coli*	6 (9.7)	–
*Citrobacter freundii*	1 (1.6)	–
*Klebsiella* spp.	1 (1.6)	–
*Rahnella aquatilis*	1 (1.6)	–
*Ralstonia picketti*	1 (1.6)	–
Unknown	3 (4.8)	–
Anaerobic bacteria		
*Staphylococcus saccharolyticus*	1 (1.6)	–
*Propionibacterium granulosum*	1 (1.6)	–
Unknown gram-positive cocci	2 (3.2)	–
Total	62 (100)	34 (100)

Growth of more than one bacterium was also common among samples taken from the control group (70 %). The only microorganisms isolated were Gram-positive cocci: *S. epidermidis* and other CNS (in all tested samples), *S. aureus* (in 15 % of samples) and *Streptococcus mitis* (in 15 % of samples).

All isolated aerobic bacteria were used to assess biofilm formation (Table 3). According to the microtiter plate method, from the 58 isolates obtained from patients with CRS, most of them were weak or moderate producers, while 5 isolates were considered to be strong biofilm producers (*E. coli*—2 strains; *S. epidermidis*—2 strains; *C. freundii*).

**Table 3:** Detection of biofilm formation by the microtiter plate method

Biofilm formation	No. of isolates (%)	
	Study group	Control group
None	11 (19.0)	4 (11.8)
Weak	30 (51.7)	13 (38.2)
Moderate	12 (20.7)	17 (50.0)
High	5 (8.6)	0 (0)

Among the strains isolated from the control group, most were moderate or weak producers. There were no strongly biofilm-forming isolates.

Using SEM, morphologic criteria described in the literature, as well as biofilm photograph examination of the tissue samples, revealed evidence of biofilm in 23 (76.7 %) specimens of the 30 patients with CRS, and in 9 (45.0 %) of the 20 septoplasty patients. This is statistically significant according to Fisher test, *p* value 0.03522.

**Fig. 1:**
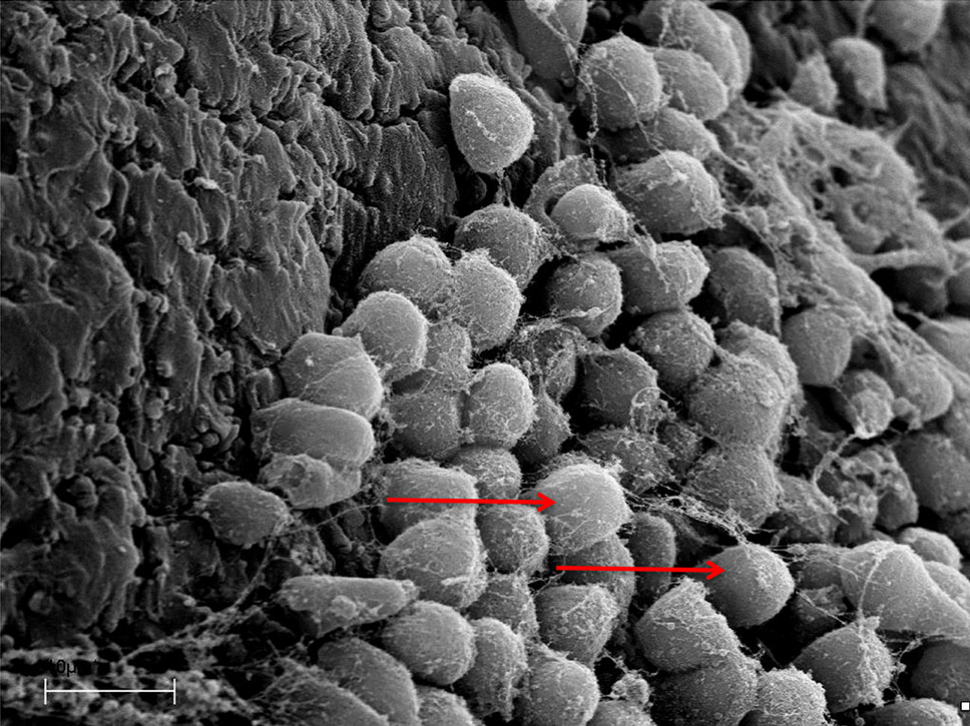
Mucosal sample obtained during paranasal sinus surgery. Note the unciliated *cylindrical* epithelium

**Fig. 2:**
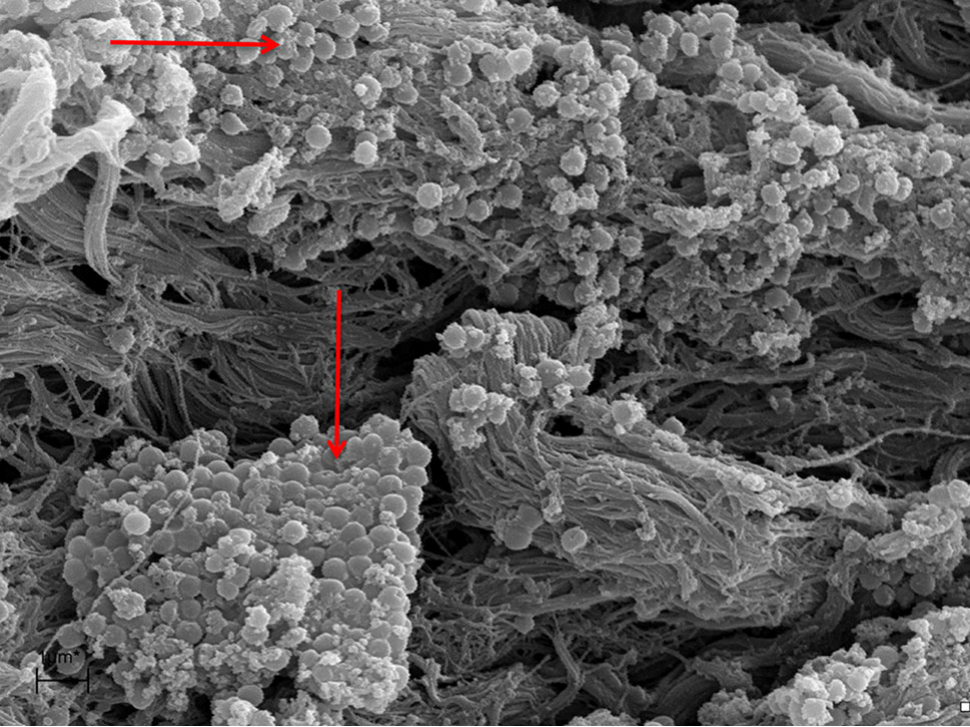
Mucosal sample obtained during paranasal endoscopic surgery. Note the presence of numerous colonies of bacterial cells (*arrows*)

**Fig. 3:**
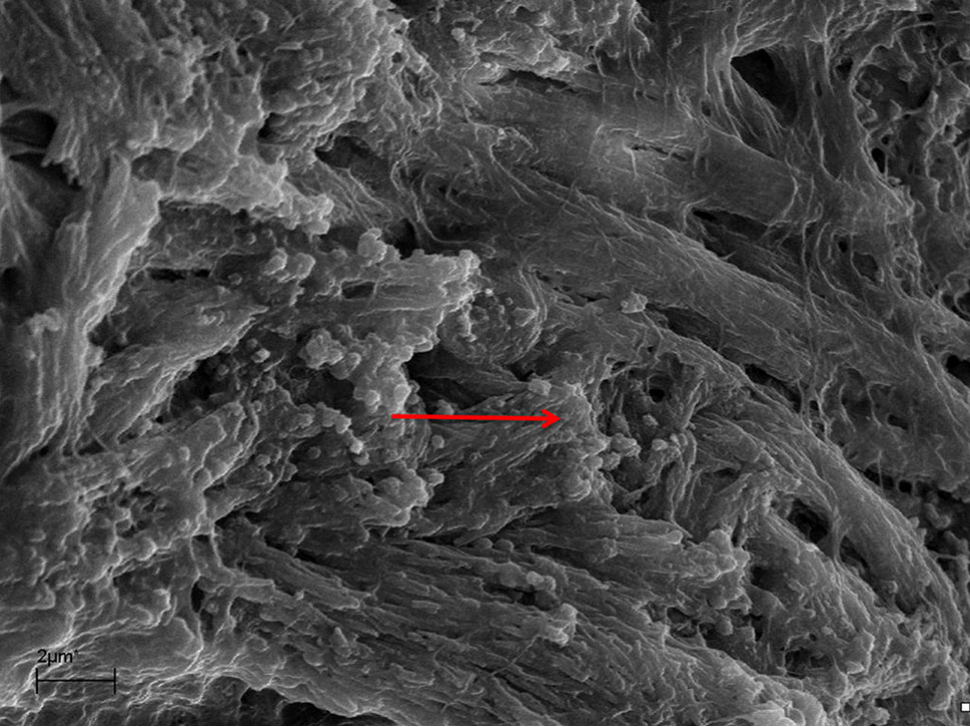
Mucosal sample obtained during paranasal endoscopic surgery. Note the three-dimensional structure, along with cells covered with extracellular matrix

**Fig. 4:**
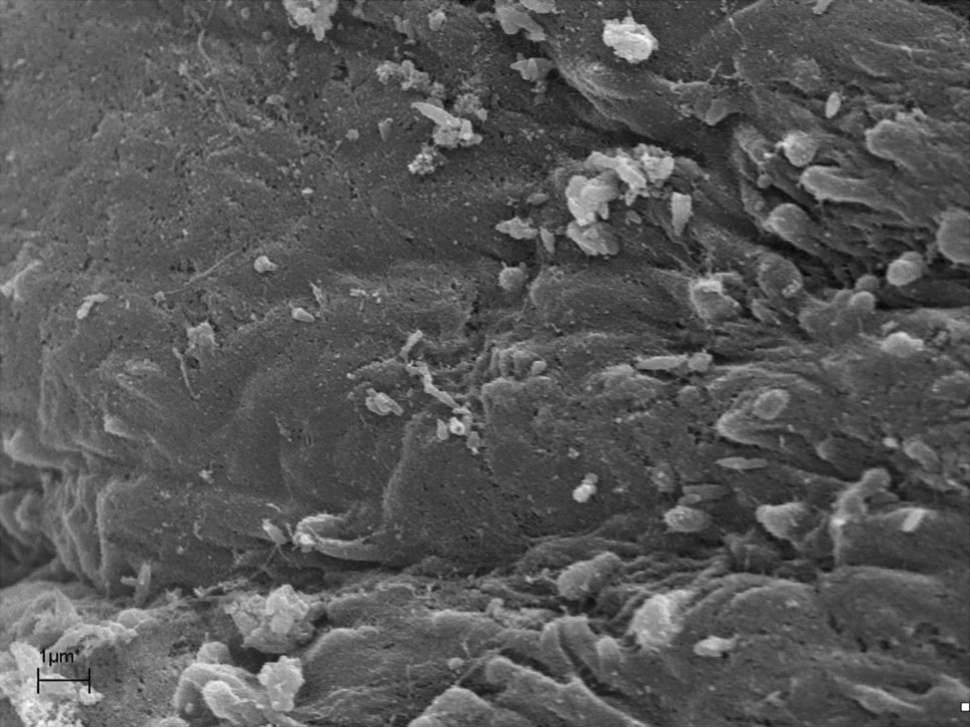
Mucosal sample obtained during paranasal endoscopic surgery. Note the absence of characteristic biofilm structures

A marked destruction of the epithelium was observed with variation in degrees of severity, from disarrayed cilia to the complete absence of cilia (Fig. 1). Biofilms were identified at different stages of the biofilm life cycle. Biofilm findings, such as water channels, three-dimensional structures, and matrix embedding elements resembling bacteria, were noted (Figs. 2, 3). In one sample, in which no microbiological growth was observed, the presence of a slightly denuded epithelium with a lack of cilia was found (Fig. 4).

## Discussion

Chronic inflammation of paranasal sinuses is one of the most common reasons for visits to a doctor’s surgery. This particular infection affects about 13 % of the general population of the United States. Unfortunately, despite the utilization of state of the art surgical and preventive treatment methods, a group of patients exhibit infections which are resistant, recurrent, and unresponsive to treatment. Frequent utilization of computer tomography and endoscopic imaging of the nasal cavity allows for the early recognition and enhanced visualization of characteristic changes caused by chronic sinusitis.

The treatment of patients with chronic sinusitis is a challenge for ENT doctors, because of the many factors that are responsible for the pathophysiology of this disease. Chronic sinusitis has recently been divided into two subgroups: chronic rhinosinusitis with polyps and without polyps. According to the literature, chronic rhinosinusitis with polyps can present not as a single disease entity, but as nasal symptoms of many different diseases [11]. Environmental factors include numerous microorganisms, and the interactions between hosts are responsible for the symptoms of chronic sinusitis. This also suggests the impact of genetic factors on the development of this disease. The presence of biofilms in patients with CRS is responsible for poor results after surgical treatment with FESS.

Our study shows that 76.67 % (23/30) of patients having FESS for CRS had evidence of biofilms on SEM micrographs. These findings correlate with the recent studies of CRS mucosal results obtained by Ramadan et al. [12, 13], Sanclement et al. [14], and Ragab et al. [15]. Sanderson et al. [16] detected bacterial biofilms in 14/18 (78 %) patients.

Microbiological testing, on the other hand, is performed only in complicated cases [17]. In healthy individuals, paranasal sinuses are not sterile [18]. In the case of recurrent episodes of sinusitis, sinuses have a tendency to become colonized by various types of microbes. Therefore, it is now thought that the etiopathological factor playing a role in the development of chronic sinusitis could be a bacterial biofilm.

On the other hand, in our material, we observed biofilm formation in 45 % of control patients, as in the literature [11]. Due to the presence of biofilm in the group of patients without evidence of chronic inflammation, the role of biofilms in the etiology of chronic sinusitis should be re-evaluated. Perhaps the role of biofilm with the associated damage to the epithelium and metaplasia in patients with CRS is a little exaggerated, because it also occurs in the control group. Similarly, Galli et al. [19] paid attention to epithelial damage at the site of bacterial biofilm development, but they also found the presence of biofilm in the ciliated epithelium. Currently, the correlation between biofilm and inflammation of the mucous membrane is still not entirely known. Some authors suggest that the Th1 inflammatory profile is responsible for biofilm, and, in turn, the Th2 inflammatory profile is responsible for inflammation of the paranasal sinuses.

With the use of in situ hybridization, Psaltis et al. [20] reviewed 38 cases of CRS and detected bacterial biofilm on the sinus mucosa of 18 patients (44 %). The discrepancy in the above-mentioned results might actually exist, or could be a result of the different detection methods used and/or differences in the patient populations studied. Furthermore, this inconsistency of data could be due to the fact that the collection of small samples was not representative of the entire sino-nasal cavity, or the fact that the biofilms were sheared off and removed with the microscopic preparation.

Regardless of these discrepancies, the consistent demonstration of biofilms on the sino-mucosal samples of patients with CRS suggests that these complex structures might play a role in either the pathogenesis or persistence of chronic rhinosinusitis. Recurrent paranasal sinusitis could be caused by fungi, and aerobic, anaerobic, gram-positive and -negative microbes. Our results revealed that among the microbiological samples obtained from CRS patients during the FESS and the control group, the most often isolated microorganism was *S. epidermidis* (37.1, 41.2 %). Other coagulase-negative cocci (CNS) accounted for 16.1 % in patients with CRS and 41.2 % in control patients of all isolated species. Furthermore, the presence of CNS was revealed in 83.3 % of infected patients. In comparison to the results obtained by other authors, our results are significantly higher. Nigro et al. [21] isolated CNS in 12.1 % of patients, whereas Mantovani et al. [22, 23] isolated *S. epidermidis* in 13.9 % of CRS patients. The role of CNS in pathogenesis is still unclear, because coagulase-negative cocci tend to colonize the nasal cavity under normal circumstances and are thought to be a contamination. Therefore, this fact refutes their potential as a constitutive factor in the development of chronic sinusitis. CNS plays an important role in the infectious process due to the formation of various intra- and extracellular biochemical compounds, such as lipopolysaccharides, and proteinaceous adhesive substances enable the successful colonization and persistence of infection [21, 22]. A comparison of the microbiological flora of the sinus mucosa between CRS and control patients revealed that *S. aureus* was more frequently present (11.3 %) in CRS patients. However, the same level of *S. epidermidis* was similar. The increased level of *S. aureus* in the disease group has important clinical implications [23].

Many authors claim that in those patients in whom traditional treatment methods are not effective, the main reason for this is gram-negative bacterial species. Hsu et al. [24], Nadel et al. [25], and Araujo et al. [26] observed gram-negative microbes in 327 (26 %) infected patients, whereas Mantovani et al. [23] observed gram-negative microbes in 58.6 % of infected patients. The predominant isolates in these studies were *P. aeruginosa* and *H. influenzae.* Our data, on the other hand, showed that the most commonly isolated gram-negative microorganism was *E. coli*, which was present in 6 patients (6/30). Mixed flora were present in 80 % of all specimens.

Within the last decade, investigation of the nature and importance of biofilm has significantly intensified. The development of new methods, such as confocal scanning laser microscopy, fluorescence in situ hybridization, and immunohistological methods, allows not only for the improved visualization of biofilm structure, but also provides us with the opportunity to identify the microbiological communities comprising biofilms. The presence of biofilm was found both in patients with CRS and in the control group.

## Conclusion

This suggests that biofilm as a single agent is not responsible for the manifestation of chronic sinusitis. It seems that a parallel correlation with other etiopathogenetic factors is necessary.
